# Validation of a stability-indicating HPLC-UV method for the quantification of acetazolamide in Oral-Mix and Oral-Mix SF

**DOI:** 10.1016/j.mex.2020.100844

**Published:** 2020-02-27

**Authors:** Charlotte Gillium, Mihaela Friciu, Nicolas Abatzoglou, Grégoire Leclair

**Affiliations:** Platform of Biopharmacy, Faculty of Pharmacy, Université de Montréal, Montréal, Canada

**Keywords:** HPLC-UV, Acetazolamide, Oral suspension, Stability, Compounding, Extemporaneous preparation

## Abstract

This manuscript details the modifications made to the HPLC assay method described in the USP monograph for Acetazolamide Compounded Oral Suspension.

• The method was modified to allow the quantification of acetazolamide in two new suspension vehicles: Oral Mix and Oral Mix SF;
• It was validated for linearity, accuracy, precision and specificity;
• It was demonstrated stability-indicating and suitable for use in a stability study using these vehicles.

## Graphical abstract

**Figure:**
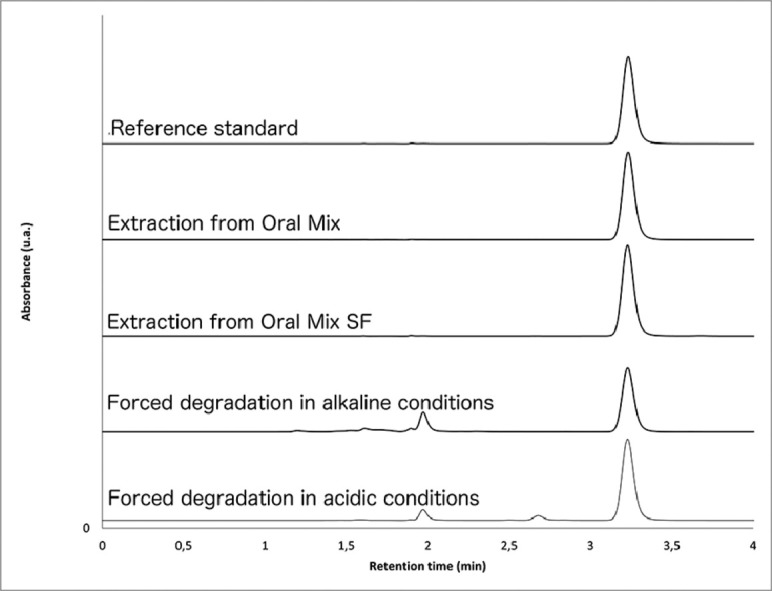

**Table:** Specification Table

Subject Area:	Pharmacology, Toxicology and Pharmaceutical Science
More specific subject area:	Oral suspensions, compounding, extemporaneous preparations, stability studies
Method name:	Stability-indicating HPLC-UV method for acetazolamide in Oral-Mix and Oral-Mix SF
Name and reference of original method:	United States Pharmacopeia 42 — National Formulary 37: Acetazolamide Oral Suspension. Rockville (MD): United States Pharmacopeial Convention; 2018.
Resource availability:	*No other resource is available.*

## Method details

### Overview

Acetazolamide is a drug indicated for the treatment of edema, epilepsy, mountain sickness and glaucoma [1], [2], [3]. It is available in the USA and in Canada in various solid oral dosage forms [4,5]. When a liquid oral dosage form is required, it can be prepared extemporaneously according to the USP monograph for Acetazolamide Compounded Oral Suspension [6]. New liquid oral acetazolamide formulations were developed using Oral Mix and Oral Mix SF suspension vehicles and a stability-indicating method was required in order to conduct a stability study for these new formulations. This manuscript details the modifications made to the USP methods for the quantification of acetazolamide in Oral Mix and Oral Mix SF vehicles. It also details the validation of this new method.

## Materials and method

### Materials

Acetazolamide bulk powder (lot 602553/B, exp. May 2019), Oral Mix (Lot I185/A, exp. January 2018), Oral Mix SF (Lot H1136, exp. October 2017) were graciously provided by Medisca Pharmaceutique Inc., Montreal, QC, Canada.

Methanol (HPLC grade, lot 144689), acetonitrile (HPLC grade, lot 141693), hydrochloric acid aqueous solution (1 M, lot 160432), sodium hydroxide aqueous solution (1 M, lot 143309), hydrogen peroxide aqueous solution (3%, lot 135515) were purchased from Fisher Scientific, Ottawa, ON, Canada.

Heptafluorobutyric acid (HFBA, lot A2716) was purchased from Santa Cruz Biotechnology, Dallas, TX, USA.

Water was purified using a Milli-Q Synthesis A10 system, Millipore, Etobicoke, ON, Canada.

Mobile phase A consisted of an aqueous solution of HFBA (0.15%) and mobile phase B was an acetonitrile solution of HFBA (0.15%).

All materials were used without further purification.

### Instrumentation

The HPLC system (Prominence UFLC, Shimadzu, Laval, QC, Canada) consisted of a SIL-20AC HT refrigerated autosampler, a DGU-20A5 solvent degasser, a LC-20AD binary pump, a CTO-20AC column oven and an SPD-M20A photodiode array detector. The HPLC column was a Zorbax SB-C18 (4.6 × 150 mm, 5 µm, P/N 883975-902, S/N USCM010053, Agilent, Santa Clara, CA, USA).

### Sample preparation

Acetazolamide suspensions were prepared from acetazolamide bulk powder using either Oral Mix or Oral Mix SF at a concentration of 25 mg mL^−1^ in a mortar using a pestle.

Samples for HPLC injections were prepared by diluting the acetazolamide suspension (50 µL) using methanol (450 µL) in a 1.5-mL centrifuge tube. The mixture was vortexed (20 s) and then centrifuged (9400 g, 10 min). Supernatant (20 µL) was further diluted using mobile phase A (480 µL), vortexed (20 s) and transferred to a sealed 96-well plate. These solutions for injection had a nominal concentration of 100 µg mL^−1^ and were analysed immediately after preparation.

### HPLC-UV conditions

The injection volume was 10 µL and all injections were conducted in duplicate. A variation of more than 0.5% between replicates triggered an investigation to identify instrumental malfunction.

The autosampler was refrigerated (5 °C) and the column oven was heated to 40 °C.

A flow gradient was used at a constant flow rate (1.0 mL min^−1^): 0 min (mobile phase A, 88: mobile phase B, 12), 4 min (88: 12), 4.5 min (70: 30), 5.5 min (70: 30), 6 min (88: 12), 13 min (88: 12).

Detection wavelength and retention time were 265 nm and 3.3 min, respectively.

## Method validation

In order to define the validation parameters of this method, including (1) Linearity and range, (2) accuracy, (3) precision and (4) specificity, several reports and guidelines pertaining to the validation of stability-indicating HPLC methods were considered [7], [8], [9], [10], [11], [12], [13], [14], [15].

Limits of detection and quantification were not formally evaluated as the lower concentration of the validation range was clearly above the limit of quantification based on signal to noise ratio. This method is not intended to be used outside of its validated range.

Since robustness was not evaluated, this method requires calibration i. Furthermore, the injection of quality control samples every 20 test samples will confirm the stability of the system throughout injection runs. This stability check was applied when assessing the stability of acetazolamide oral suspensions over a period of 90 days***.***

### Linearity and range

Acetazolamide suspensions (25 mg mL^−1^) were prepared in Oral Mix and Oral Mix SF as described above. These suspensions (500 µL) were diluted using methanol (4.5 mL), vortexed (20 s) and then centrifuged (9400 g, 10 min). Samples (20, 40, 60, 80 and 120 µL) of the supernatant (2.5 mg mL^−1^) were further diluted using mobile phase A (1980, 1960, 1940, 1920, 1880 µL) and vortexed (20 s) to obtain standard solutions at concentrations of 25, 50, 75, 100 and 150 µg/mL prior to injection in triplicate.

HPLC response was plotted as a function of acetazolamide concentration for both Oral Mix and Oral Mix SF. A linear regression was calculated for both plots (*y*-intercept was forced through zero). Coefficient of determination were respectively 0.9997 for Oral Mix and 0.9995 for Oral Mix SF.

The target concentration of test samples is 100 µg mL^−1^. The range of this method is 25–150 µg mL^−1^, or 25–150% of the target concentration.

### Accuracy

Standard solutions of acetazolamide in mobile phase A were prepared at concentrations of 25, 50, 75, 100 and 150 µg mL^−1^ and analysed using the HPLC-UV method. As listed in Table 1, in order to evaluate the effects of the matrix as well as sample preparation, the areas of the acetazolamide peaks were compared to the areas obtained during the validation of linearity and range described above. Furthermore, the concentration of acetazolamide of these samples was back calculated using the linear regression slope parameter and compared with their nominal value to establish the accuracy of the linear regression at all tested concentrations.

**Table 1:** Accuracy.

Nominal conc. (µg mL^−1^)	HPLC response in vehicle	HPLC response in mobile phase	Recovery vehicle:mobilephase (%)	Back calculated conc. (µg mL^−1^)	Back calculated recovery (%)
**Acetazolamide in Oral Mix**					
25	714288	698669	102.2	24.6	98.4
50	1447021	1398513	103.5	49.8	99.6
75	2141535	2099685	102.0	73.7	98.3
100	2954532	2808259	105.2	101.7	101.7
150	4345057	4214417	103.1	150.0	100.0
**Acetazolamide in Oral Mix SF**					
25	699705	698669	100.1	24.4	97.6
50	1417084	1398513	101.3	49.5	99.0
75	2137448	2099685	101.8	74.6	99.5
100	2806614	2808259	99.9	98.0	98.0
150	4347873	4214417	103.2	151.8	101.2

The effect of sample preparation and matrix on the accuracy of the method resulted in recoveries ranging from 99.9% to 105.2% at all tested concentrations over the investigated range. Furthermore, the back calculated recovery varied from 97.6% to 101.7% at all tested concentrations over the method range.

### Precision

Intraday precision corresponds to the coefficient of variation of the triplicated analyses performed for the validation of linearity and range detailed above. Interday variability is similarly calculated from analyses performed on three different days. These results are reported in Table 2.

**Table 2:** Precision.

Nominal conc. (µg mL^−1^)	Intraday CV (%)	Interday CV (%)
**Acetazolamide in Oral Mix**		
25	0.17%	1.34%
50	0.14%	2.31%
75	0.07%	2.03%
100	0.05%	1.46%
150	0.09%	1.92%
**Acetazolamide in Oral Mix SF**		
25	0.05%	4.75%
50	0.03%	3.23%
75	0.05%	2.85%
100	0.02%	3.76%
150	0.12%	3.39%

The precision of the method evaluated as the intraday CV and the interday CV were not more than 0.17% and 4.75%, respectively, at any of the tested concentrations over the method range.

### Specificity

An acetazolamide suspension (25 mg mL^−1^) was prepared in Oral Mix as described above. Aliquots of this suspension (0.5 mL) were mixed with water (0.5 mL), aqueous hydrogen peroxide 3% (0.5 mL), aqueous hydrochloric acid 1 M (0.5 mL) and aqueous sodium hydroxide 1 M (0.5 mL). These four solutions were stored for 3 h at 60 °C. The acidic solution (100 µL) was neutralized using aqueous sodium hydroxide 1 M (50 µL) and diluted using methanol (350 µL). Similarly, the alkaline solution was neutralized using aqueous hydrochloric acid 1 M (50 µL) and diluted using methanol (350 µL). The water and peroxide solutions were directly diluted using methanol (400 µL). All these solutions were vortexed (20 s) and centrifuged (9400 g, 10 min). Supernatants (20 µL) were recovered and diluted in mobile phase A (480 µL) to achieve a nominal concentration of 100 µg mL^−1^ (prior to degradation) and analysed using the HPLC-UV method. The chromatograms obtained from these analyses were compared to the chromatograms obtained from acetazolamide suspensions in Oral Mix and Oral Mix SF (25 mg/mL) submitted to sample preparation for HPLC injection. They were also compared to the chromatograms obtained from a solution of acetazolamide in mobile phase A (100 µg/mL).

**Fig. 1:**
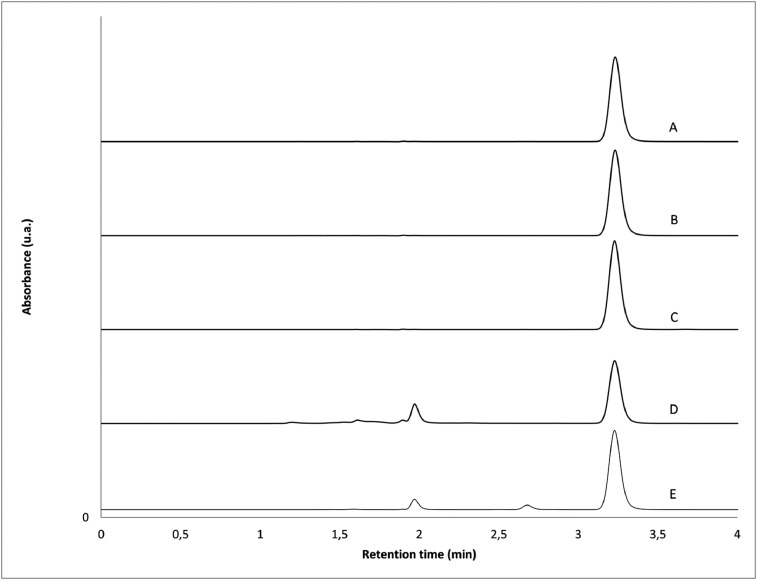
Representative chromatograms. (A) Acetazolamide standard solution in an aqueous solution of HFBA 0.15% (mobile phase A); (B) acetazolamide suspension prepared from bulk drug using Oral Mix vehicle; (C) acetazolamide suspension prepared from bulk drug using Oral Mix SF; acetazolamide suspension in mobile phase A after either alkaline (D, NaOH 0.1%, 60 °C, 3 h) or acidic (E, HCl 0.1%, 60 °C, 3 h) degradation. Degradation in water and peroxide did not yield any additional peak (data not shown).

Recovery values of 102%, 103%, 73% and 92% were observed following degradation in water, peroxide, alkaline and acidic conditions, respectively. As shown in Fig. 1, no peak overlap of acetazolamide with excipients, impurities or degradation products was observed. Acetazolamide peak purity index calculated between 235 and 295 nm was not less than 0.9999 in all cases.

## Conclusion

A stability-indicating method for the quantification of acetazolamide in Oral Mix and Oral Mix SF suspensions was developed and validated. This method is intended to be used for the evaluation of the stability of acetazolamide in these two oral suspension vehicles. This methods improves the USP assay method for acetazolamide compounded oral suspension. The USP method was developped and validated for these two vehicles: 1:1 mixture of Vehicle for Oral Solution, NF (regular or sugar-free), and Vehicle for Oral Suspension, NF, or Cherry Syrup, NF. The method reported in this manuscript was specifically developped and validated for acetazolamide compounded oral formulation in two other slightly different commercial vehicles: Oral Mix and Oral Mix SF. On the one hand, the latter method used a lower injection volume (10 vs. 20 µL) and a gradient elution system wich allowed sharper peaks with a better resolution. On the other hand, the USP method provided stronger signals and a simpler isocratic elution system.
